# Glucose-Regulated Protein 78 Is a Potential Serum and Imaging Marker for Early Detection of Ovarian Cancer

**DOI:** 10.3390/cancers15041140

**Published:** 2023-02-10

**Authors:** Elizabeth A. Paris, Janice M. Bahr, Jacques S. Abramowicz, Sanjib Basu, Animesh Barua

**Affiliations:** 1 Department of Anatomy & Cell Biology, Rush University Medical Center, Chicago, IL 60612, USA; 2 Department of Animal Sciences, University of Illinois at Urbana-Champaign, Urbana, IL 61801, USA; 3 Department of Obstetrics and Gynecology, University of Chicago, Chicago, IL 60637, USA; 4 Department of Internal Medicine, Rush University Medical Center, Chicago, IL 60612, USA; 5 Department of Pathology, Rush University Medical Center, Chicago, IL 60612, USA; 6 Department of Obstetrics and Gynecology, Rush University Medical Center, Chicago, IL 60612, USA

**Keywords:** chronic inflammation, early detection, glucose-regulated protein 78, laying hen, ovarian cancer, oxidative stress, targeted-imaging agents, transvaginal ultrasound imaging

## Abstract

**Simple Summary:**

Ovarian cancer (OVCA) is a fatal gynecological disease for which there is no early detection test. Glucose-regulated protein 78 (GRP78), a protein marker of stress, increases during chronic stress. Chronic stress has been suggested as a hallmark of cancer development. This study examined whether expression of GRP78 is associated with development of OVCA and whether GRP78 can predict OVCA at early stage. This study found GRP78 expression and its secretion in blood increased during OVCA development and progression. This study also developed a GRP78-targeted ultrasound scanning agent that detected ovarian tumors at early stages. Thus, a woman with high levels of GRP78 in her blood may be referred to have targeted-ultrasound scanning for confirming if she has ovarian tumors. These results will be a foundation for a clinical study to examine the feasibility of GRP78 as a potential marker of blood and ultrasound scanning for early detection of OVCA.

**Abstract:**

Background: Understanding malignant transformation associated with ovarian cancer (OVCA) is important to establish early detection tests. This study examined whether expression of glucose-regulated protein 78 (GRP78, marker of cellular stress) increases during OVCA development, and whether GRP78 can be detected by targeted-transvaginal ultrasound (TVUS) imaging. Methods: Normal ovaries (*n* = 10), benign (*n* = 10) and malignant ovarian tumors at early (*n* = 8) and late stages (*n* = 16), hens with and without ovarian tumors at early and late stages (*n* = 10, each) were examined for GRP78 expression during OVCA development by immunohistochemistry, immunoblotting, gene expression and immunoassay. Feasibility of GRP78-targeted TVUS imaging in detecting early OVCA was examined. Results: Compared with normal ovaries and benign tumors, intensity of GRP78 expression was higher (*p* < 0.0001) in OVCA patients. Compared with normal (9007.76 ± 816.54 pg/mL), serum GRP78 levels were significantly higher (*p* < 0.05) in patients with early (12,730.59 ± 817.35 pg/mL) and late-stage OVCA (13,930.12 ± 202.35) (*p* < 0.01). Compared with normal (222.62 ± 181.69 pg/mL), serum GRP78 levels increased (*p* < 0.05) in hens with early (590.19 ± 198.18 pg/mL) and late-stage OVCA (1261.38 ± 372.85) (*p* < 0.01). Compared with non-targeted, GRP78-targeted imaging enhanced signal intensity of TVUS (*p* < 0.0001). Conclusions: Tissue and serum levels of GRP78 increase in association with OVCA. GRP78 offers a potential serum and imaging marker for early OVCA detection.

## 1. Introduction

Approximately 90% of all ovarian cancers (OVCA) are epithelial, and type-II OVCA is the most lethal form which, in most cases, is detected at stage-III and stage-IV [1,2]. The 5-year survival rate of OVCA patients when detected at late stages is approximately 45%, as opposed to >90% when detected at early stage [3]. OVCA recurs frequently [4], so an effective early detection test for OVCA is urgently needed. Serum levels of cancer antigen 125 (CA-125) with or without traditional transvaginal ultrasound (TVUS) imaging, are the currently used methods for OVCA detection. CA-125 is not specific for early-stage OVCA, as its serum level is also elevated in ovarian or non-ovarian benign conditions, and limited resolution of traditional TVUS cannot detect early changes associated with OVCA. Further, CA-125 alone or in combination with TVUS did not improve the rates of early detection of OVCA [5]. In addition, no target(s) in the ovary indicative of malignant transformation has been established for TVUS imaging. Therefore, effective serum markers, imaging target(s) in the ovary and an imaging agent to detect this target need to be developed to establish an early detection test for OVCA [2].

Chronic inflammation and oxidative stress have been suggested as risk factors for malignant development [6]. Ovaries and fimbriae of the fallopian tubes are exposed to various internal and external inflammatory factors, including ovulation and/or infection [7]. Ovulation is followed by the influx of immune cells and frequent ovulation leads to chronic inflammation and oxidative stress [7,8,9]. Long-term exposure to oxidative stress increases production of glucose-regulated protein 78 (GRP78), an endoplasmic reticulum (ER) resident protein and marker of oxidative stress. To withstand and survive from stressful conditions, GRP78 is expressed by the cell surface and secreted into the circulation [10,11,12]. Thus, GRP78 has the potential to be used for the detection of OVCA. The goals of this study were to examine systematically: (1) whether expression of GRP78 increases at cellular and molecular levels during OVCA development, and (2) whether GRP78 can be detected by TVUS imaging. As access to patients with early-stage OVCA is difficult, the study on GRP78-targeted TVUS imaging was performed in laying hens.

Rodents do not develop OVCA spontaneously, and induced OVCA in rodents does not represent spontaneous OVCA in humans. In contrast, laying hens (*Gallus gallus domesticus*) develop OVCA spontaneously with high incidence rates and remarkable similarities to human OVCA [13,14,15,16]. Ovarian tumors in hens can also be detected using an ultrasound scanner similar to that used in the clinic [17]. Three experiments were conducted in this study, including the determination of GRP78 expression in normal or OVCA patients (experiment 1) or hens (experiment 2). In the third experiment, a GRP78-targeted imaging agent was developed, and its feasibility for OVCA detection by GRP78-targeted TVUS imaging was examined.

## 2. Materials and Methods

### 2.1. Experiment 1: Clinical Samples

Normal ovarian and fimbrial (*n* = 10) tissues and BRCA+ fimbrial tissues (*n* = 10) were obtained from subjects who underwent surgery for hysterectomy due to non-ovarian reason. Ovarian benign tumors (*n* = 10, including serous cystadenomas = 3 and cystadenofibromas = 3) or high-grade serous carcinoma (HGSC) at early (*n* = 8) and late stages (*n* = 16) were obtained from the Department of Pathology, Rush University Medical Center (RUMC) following surgery. Staging and histological types of tumors were obtained during surgery and from final pathology reports. In addition, ovaries with endometriosis (*n* = 3) were also used as an additional control with benign condition. Blood samples from subjects and patients were collected from Pathology following surgery. Serum was separated and stored at −80 °C for use in immunoassay. Specimens were examined by routine staining, immunohistochemistry (paraffin sections, 5 µm thick), proteomic and molecular biological studies (mRNA expression).

### 2.2. Experiment 2: Preclinical Specimens

White Leghorn laying hens were reared under standard poultry husbandry practices. Three to four-year-old hens (*n* = 190) were scanned with traditional TVUS as reported earlier [17]. The number of hens for control (healthy) and OVCA (experimental) groups were determined to yield a true between-group difference in their tissue and/or serum level of GRP78. Accordingly, healthy hens with normal ovaries (*n* = 10) or hens with ovarian tumors at early (*n* = 10, serous = 4, endometrioid = 3, mucinous = 3) or late stages (*n* = 10, serous = 4, endometrioid = 4, mucinous = 2) were used. Blood was collected, hens were euthanized and examined for presence of solid masses, and the extent of dissemination of OVCA was recorded. Normal and tumor tissues were processed for immunohistochemistry, protein and gene expression studies. Serum was separated and stored at −80 °C for later use.

### 2.3. Experiment 3: Development of GRP78-Targeted Imaging Agents and Targeted-TVUS Imaging of Hens

Three to four-year-old laying hens (*n* = 50) with or without tumors were selected by traditional TVUS imaging as mentioned earlier [17]. GRP78-targeted imaging agents were developed using anti-GRP78 antibodies (Abcam, Cambridge, MA, USA) as reported earlier, with little modification [18]. Briefly, anti-GRP78 antibodies were biotinylated using biotinylation kit (Abcam, Cambridge, MA, USA) and then conjugated with microbubbles containing streptavidin (Targeson, Inc., San Diego, CA, USA). Hens were scanned with TVUS imaging before and 5 min after injection with 10 µL of GRP78-targeted imaging agents per kilogram body weight of hen, as reported earlier [18]. TVUS images were archived and analyzed to determine the signal intensities of the tissues before and after injection of targeted-imaging agent and expressed as mean ± standard error of the mean (SEM) in 20 mm^2^ area of tissue. Hens were euthanized following targeted imaging, examined for the presence of OVCA, and tissues were processed.

### 2.4. Preparation of Ovarian Specimen for Biochemical Analysis

Total tissue protein, nuclear matrix protein (NMP), and total RNA were collected from all samples as reported previously [19,20,21,22]. Additionally, lysates of normal human ovarian surface epithelial cells (HOSE) and ovarian malignant cell lines including OVCAR3, SKOV3 and Caov3 (ATCC, Manassas, VA, USA) were collected and examined for GRP78 expression by immunoblotting.

### 2.5. Immunohistochemistry

Immunohistochemical detection of GRP78 expression was performed using anti-GRP78 antibodies, mentioned above, as reported previously [23]. The intensities of GRP78 expression by normal and malignant cells were determined as reported earlier [23], and expressed as the mean intensity ± standard error of the mean (SEM) in 20 mm^2^ area of the tissue. Intensities of immunohistochemical staining were reported as arbitrary values as defined by the computer-assisted imaging software (MicroSuite^TM^ version 5, Olympus American, Inc., Center Valley, PA, USA).

### 2.6. Immunoassay

Serum levels of GRP78 in representative normal and OVCA patients and hens were determined by immunoassay using commercial GRP78 ELISA kits for human and chicken, respectively, as per the manufacturer’s instruction (MyBioSource, Inc., San Diego, CA, USA). A standard curve was generated, and serum GRP78 levels were determined with reference to the standard curve as per manufacturer’s recommendation using a software program (Gen5, version 2.00, Biotek Instruments, Inc., Winooski, VT, USA). Values are presented as mean concentration ± standard error of the mean (SEM).

### 2.7. One- and Two Dimensional (1-&2-D) Western Blot (WB)

Immunohistochemical expression of GRP78 was confirmed by 1-& 2-D-WB using the same antibodies mentioned above and similarly reported earlier [23,24]. Two-dimensional gel electrophoresis was performed as reported previously [25]. Proteins were separated and transferred to a nitrocellulose membrane [26], and immunoreactive GRP78 protein on the membrane was determined as reported earlier [19].

### 2.8. Semi-Quantitative and Quantitative Real Time Polymerase Chain Reaction (qRT-PCR)

GRP78 mRNA expression was assessed by qRT-PCR assays using human-specific GRP78 primer, and the 18s SnRNA primer (an endogenous primer) was designed from Qiagen (Foster City, CA, USA). The differences in GRP78 mRNA expression levels were calculated as fold-changes as reported earlier [22,27]. Semi-quantitative RT-PCR was performed for GRP78 mRNA expression, as reported earlier [28] (data not shown).

### 2.9. Statistical Analysis

Differences in the intensities of GRP78 expression among different groups of clinical specimens, as well as hens, were assessed by ANOVA. Differences in the intensity of GRP78 protein expression (in immunoblotting), GRP78 mRNA expression or serum GRP78 levels among normal or tumor ovaries, both in women and hens, were analyzed similarly. Significant differences in the signal intensity of TVUS imaging due to GRP78-targeted imaging agent were examined using two-sample t-tests (pre-targeted vs. post-targeted imaging from OVCA hens). Correlations in tissue GRP78 protein and GRP78 gene (mRNA) expression were examined using the Pearson co-efficient of correlation at 95% confidence interval (*n* = 4). All reported *p* values are 2-sided, and *p* < 0.05 was considered significant. Statistical analyses were performed using GraphPad Prism (GraphPad software version 6, La Jolla, CA, USA).

## 3. Results

### 3.1. Expression of GRP78 in Normal Ovaries or Ovaries with Tumors in Patients

**Figure 1:**
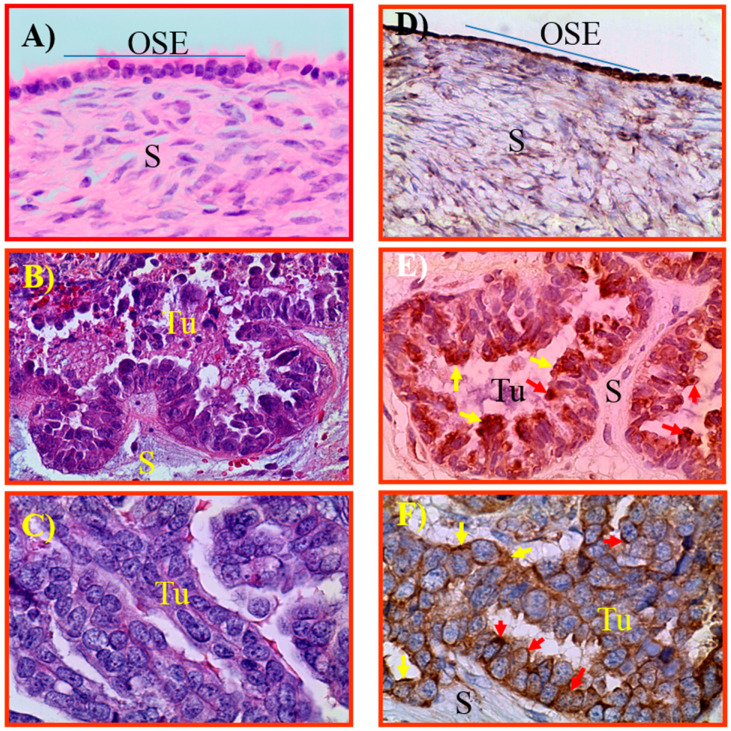
Expression of GRP78 by normal ovaries and malignant ovarian tumors at early and late stages; sections were stained with hematoxylin & eosin (**A**–**C**) and with anti-GRP78 antibodies (**D**–**F**). (**A**) Ovarian section from a normal postmenopausal ovary showing ovarian surface epithelial (OSE) cells and stroma (S). (**B**) Section of an ovarian serous malignant tumor (Tu) at early stage showing a compact sheath-like tissue mass surrounded by fibromuscular layer. (**C**) Section of an ovarian serous malignant tumor at late stage showing a papillae-like appearance of malignant cells. (**D**) Normal postmenopausal ovarian section showing GRP78 staining by the OSE cells. (**E**,**F**) Ovarian serous malignant tumors at early and late stages, respectively, showing GRP78 staining by the cell surface (yellow arrows) and nucleus (red arrows). Magnification = 40×.

Normal ovaries from women at the perimenopausal stage showed ovarian surface epithelium (OSE) with occasional presence of stromal follicles. In contrast, ovaries in normal postmenopausal women contained OSE and stromal fibroblasts with no follicles (Figure 1A). Malignant ovarian tumors, including early and late stages used in this study, were serous carcinoma (Figure 1B,C). Expression of GRP78 was detected in OSE cells in normal ovaries and in tumor cells of benign and malignant ovaries (Figure 1D–F). Similarly, fimbrial surface epithelial (FSE) cells of normal or in BRCA1+ fallopian tube, one of the sites of origin of ovarian HGSC, were examined for GRP78 expression (Supplementary Figure S1). Normal FSE showed weak expression for GRP78 while an intense expression for GRP78 was observed in the FSE of BRCA+ subjects (high risk for OVCA development). In addition to cell surface expression, malignant cells also showed cytoplasmic and nuclear staining for GRP78. Compared with normal ovaries and ovaries with benign tumors, the staining for GRP78 was stronger in HGSC. Furthermore, ovarian endometriotic lesions showed a weak expression for GRP78 (Supplementary Figure S2).

**Figure 2:**
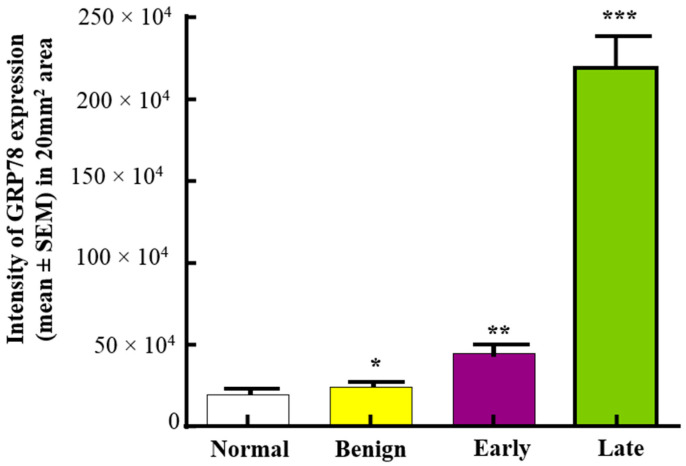
Changes in the intensity of GRP78 expression during OVCA development and progression in patients. Compared with normal ovaries and ovaries with benign tumors, expression of GRP78 was significantly higher in ovaries with early-stage HGSC (*p* < 0.001) and increased further in late-stage HGSC (*p* < 0.0001). * *p* < 0.05, ** *p* < 0.01, *** *p* < 0.0001.

Compared with normal (19.20 × 10^4^ ± 0.155 × 10^4^ in 20 mm^2^ area), the intensity of GRP78 expression was higher in benign tumors (24.03 × 10^4^ ± 0.157 × 10^4^ in 20 mm^2^). The intensity of GRP78 staining was significantly (*p* < 0.0001) higher in malignant tumors at early (44.94 × 10^4^ ± 0.48 × 10^4^ in 20 mm^2^ area) and late stages (219.2 × 10^4^ ± 18.0 × 10^4^ in 20 mm^2^ area) when compared to normal and benign tumors (Figure 2).

**Figure 3:**
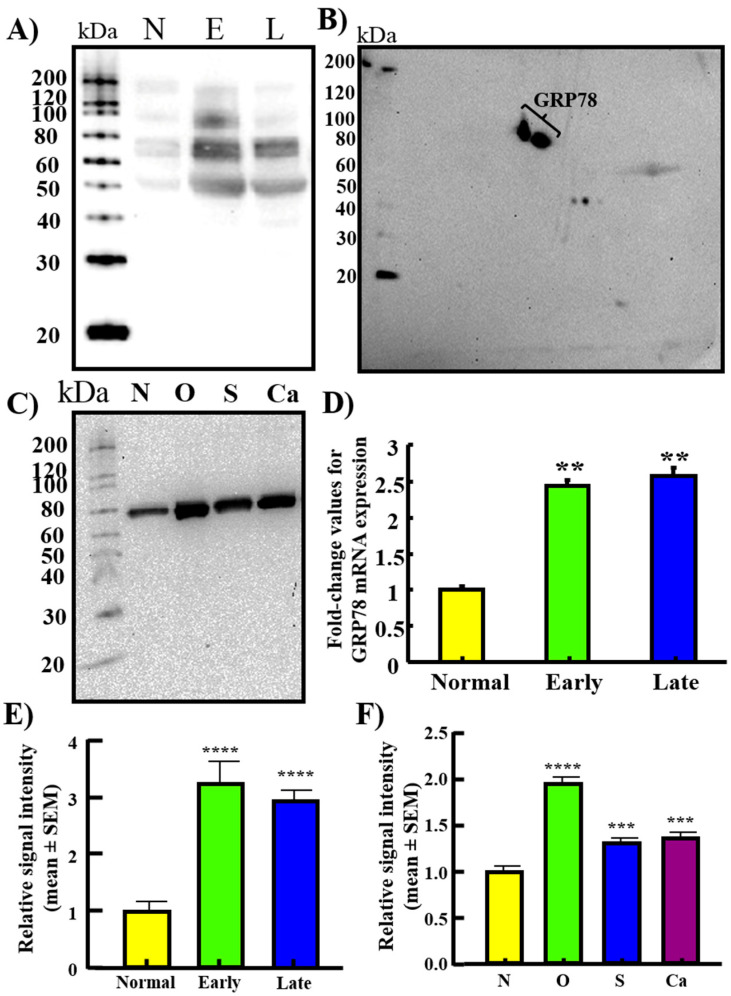
GRP78 protein and gene expression by normal ovaries and ovaries with HGSC at early and late stages. (**A**) One-dimensional Western blotting (1-D-WB) showed GRP78 expression by the normal (N), early (E) and late (L) stage OVCA (*n* = 4 each). Anti-GRP78 antibodies showed multiple immunoreactive bands including one at approximately 78 kDa with strong intensity from early and late stage tumors. Normal ovary showed very weak immunoreactive signal for GRP78 expression. (**B**) 2-D-WB of a malignant tumor (late-stage HGSC) confirmed immunoreaction for GRP78 of approximately 78 kDa. (**C**) GRP78 expression by the normal human ovarian surface epithelial cells (HOSE, N) and OVCAR3 (O), SKOV3 (S), Caov3 (Ca) ovarian cancer cell lines (*n* = 4 each). Compared to the lysate from normal cells, strong immunoreaction for GRP78 was shown by all OVCA cell lines. (**D**) Compared with normal, gene expression study showed strong amplification for GRP78 mRNA in early- and late-stage OVCA (*p* < 0.01). (**E**) Relative signal intensity of GRP78 protein expression detected by 1-D-WB bands shown in panel A presented as an intensity ratio (mean ± SEM). (**F**) Relative signal intensity of GRP78 protein expression detected in 1-D-WB bands shown in panel C presented as an intensity ratio (mean ± SEM). ** *p* < 0.01, *** *p* < 0.001, **** *p* < 0.0001.

Tissue expression of GRP78 was confirmed by 1-D- & 2-D-WB of normal and malignant ovaries. 1-D-WB showed multiple bands with one at approximately 78 kDa for GRP78. 2-D-WB confirmed the intense expression of GRP78 at 78 kDa in malignant ovaries (Figure 3A,B). In addition, 1-D-WB of cell lysates showed stronger bands for GRP78 in OVCAR3, SKOV3 and Caov3 cancer cells than normal cells (HOSE) (Figure 3C). Immunoblot showed similar patterns of signal intensities of GRP78 protein expression in cancer cells to that of immunohistochemical expression of GRP78 (Figure 3E,F). Compared with normal ovaries, quantitative assays showed significantly higher expression of GRP78 gene in malignant ovaries (*p* < 0.01). Fold changes in GRP78 gene expression (Figure 3D) were positively correlated with the intensity of GRP78 protein expression (in immunoblotting, Figure 3E) (R^2^ = 0.96, *p* < 0.05, *n* = 4). Thus, protein and gene expression data for GRP78 support immunohistochemical observations of an increase in GRP78 expression during OVCA development and progression.

### 3.2. Changes in GRP78 Expression during OVCA Development in Hens

**Figure 4:**
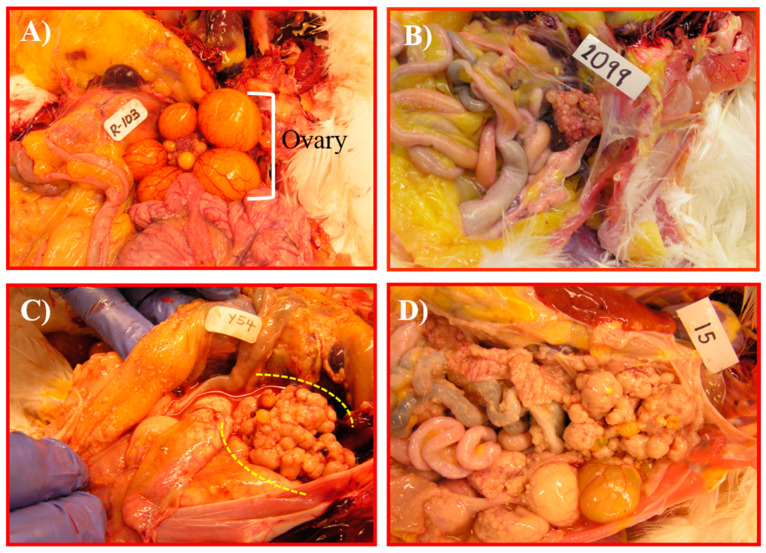
Spontaneous incidence of ovarian cancer in hens. (**A**) Ovary in a healthy laying hen showing a hierarchy of different sizes of preovulatory follicles. (**B**) A regressed ovary and oviduct in a hen; no preovulatory follicle is seen in the ovary. (**C**) OVCA at early stage in a hen. The tumor mass appears like a cauliflower (dotted lines), limited to the ovary and is accompanied with little ascites. (**D**) OVCA at late stage in a hen. Tumor mass is metastasized to other organs.

**Figure 5:**
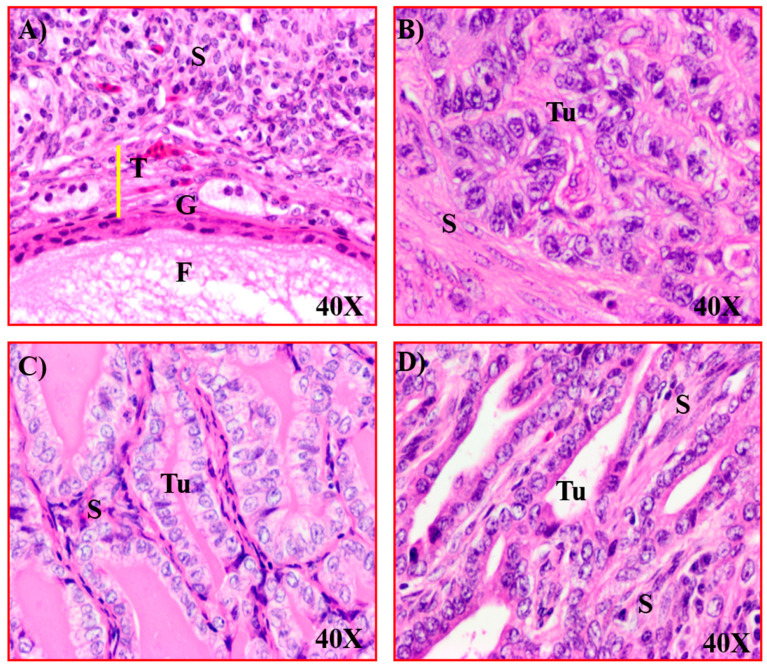
Microscopic presentation of ovarian adenocarcinomas in hens. (**A**) Section of a normal ovary in a hen showing a follicle (F) embedded in the stroma (S). (**B**) Section of a serous OVCA in a hen. The tumor (Tu) appears like a compact sheath of malignant cells surrounded by fibromuscular layers in the stroma. (**C**) Section of an ovarian mucinous carcinoma in a hen. The tumor consists of malignant cells of columnar morphology arranged as a single layer in glandular structure containing mucin-like secretion in the lumen. (**D**) Section of an ovarian endometrioid carcinoma in a hen showing back-to-back confluent tumor glands consisting of a single layer of malignant cells. G = granulosa cell layer, T = theca layer in stromal follicle, Magnification = 40×.

Normal ovaries in older (3–4 year old) hens had fewer preovulatory follicles (2–3 large follicles) (Figure 4A). In hens that had ceased laying, the ovaries became atrophied with no large follicle (Figure 4B). In the early stage of OVCA, solid masses were limited to the ovary accompanied with or without little to moderate ascites (Figure 4C). Tumors at late stage of OVCA metastasized to distal organs and were accompanied by profuse ascites (Figure 4D). Routine histological examination confirmed the presence of tumors in hens (Figure 5). Microscopically, normal ovaries in hens contained surface epithelial cells, embedded stromal follicles, stromal atretic follicles, and a few scars and remnants of regressing postovulatory follicular tissues (Figure 5A). Histological examination of tumors showed the tumors were serous, endometrioid and mucinous (Figure 5B–D).

**Figure 6:**
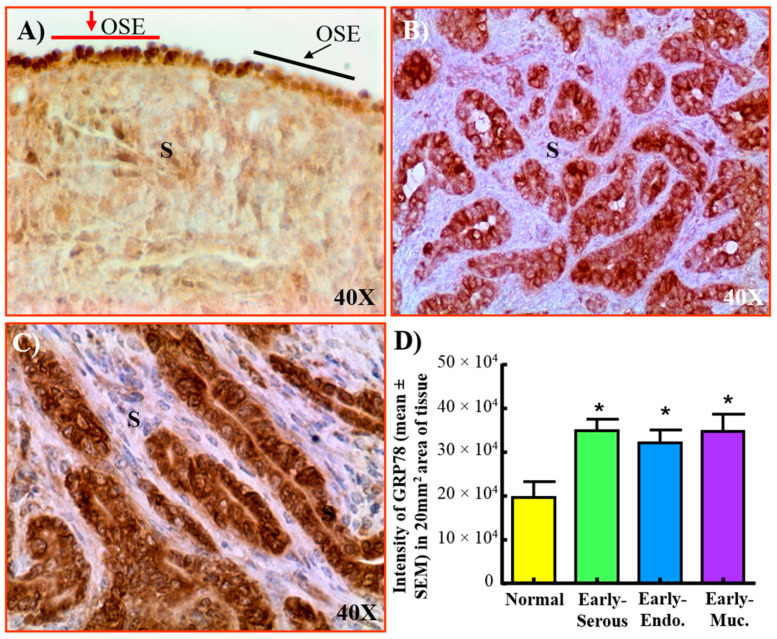
Expression of GRP78 by normal ovary and ovarian malignant tumors at early and late stages in hens. (**A**) Normal ovarian section showing GRP78 staining by few ovarian surface epithelial (OSE) cells (red arrows), while no staining was observed in others (black arrows). (**B**) Ovarian endometrioid carcinoma at early stage showing intense staining for GRP78. (**C**) Ovarian serous carcinoma at late stage showing malignant cells stained strongly for GRP78. The nucleus as well as surface of malignant cells showed strong immunoreactivity for GRP78 staining. (**D**) Compared to normal ovaries, the expression of GRP78 was significantly high in ovaries with tumor at early stage (*p* < 0.03). However, significant differences were not observed in the intensity of GRP78 expression among different histological types of OVCA (serous *n* = 4, endometrioid *n* = 3, mucinous *n* = 3). Endo = Endometrioid, Muc = Mucinous, S = Stroma, Magnification = 40×. * *p* < 0.05.

In normal ovaries, GRP78 was detected in few OSE cells (red arrow) while other OSE cells did not stain for GRP78 (black arrow, Figure 6A). Malignant cells showed intense staining for GRP78 (Figure 6B,C). Compared with normal ovaries (19.96 × 10^4^ ± 3.5 × 10^4^), the intensity of GRP78 expression was significantly (*p* < 0.03) higher in malignant tumors at early stages, including serous (34.70 × 10^4^ ± 2.58 × 10^4^), endometrioid (32.15 × 10^4^ ± 2.91 × 10^4^) and mucinous (34.60 × 10^4^ ± 4.0 × 10^4^) in 20 mm^2^ area of tissue (Figure 6D). Significant differences were not observed in GRP78 expression among different histological types of malignant tumors.

### 3.3. Serum Levels of GRP78

**Figure 7:**
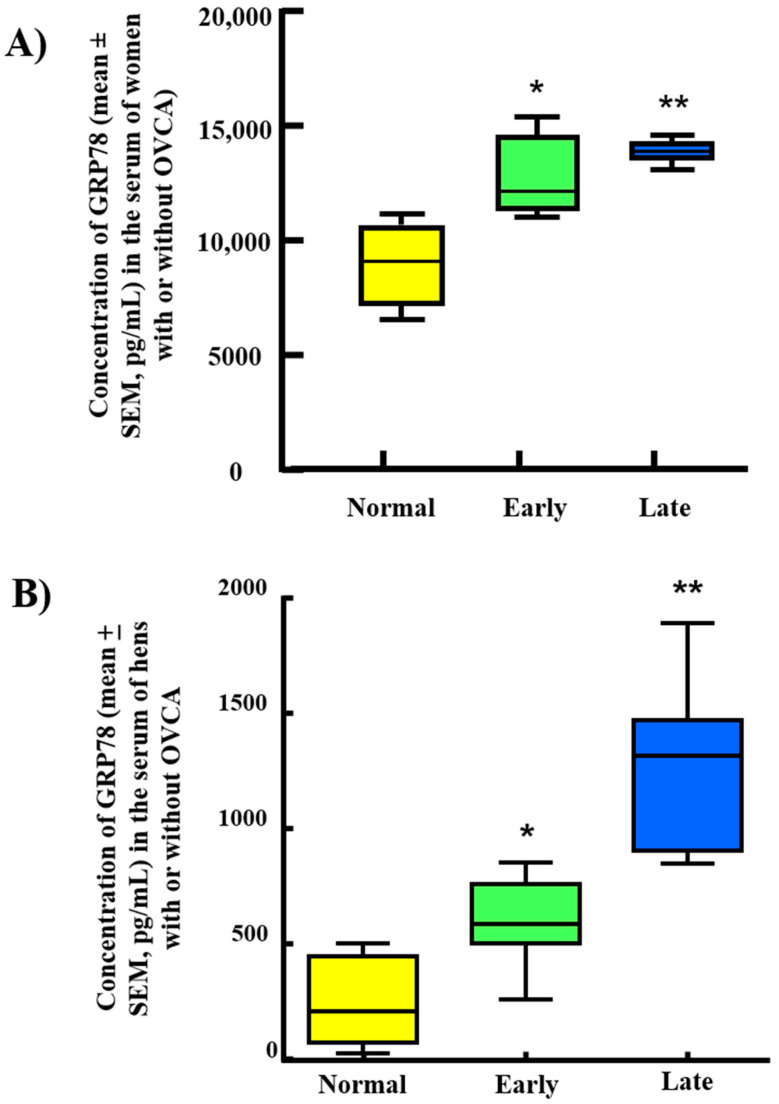
Changes in the serum expression of GRP78 during the development and progression of OVCA in patients and hens. (**A**) Box-plot graph shows the serum levels of GRP78 were significantly (*p* < 0.05) higher in patients with early-stage OVCA compared to women without OVCA. Serum level increased further in women with late-stage OVCA (*p* < 0.01). (**B**) Box-plot graph shows the serum levels of GRP78 were significantly (*p* < 0.05) higher in hens with early stage OVCA than normal hens and increased further in late-stage OVCA (*p* < 0.01). * *p* < 0.05, ** *p* < 0.01.

Compared with normal levels (9007.76 ± 816.54 pg/mL), serum levels of GRP78 were higher (*p* < 0.05) in patients with early stage (12,730.59 ± 817.35 pg/mL) and late-stage OVCA (13,930.12 ± 202.35 pg/mL) (*p* < 0.01) (Figure 7A). Compared with normal (222.62 ± 181.69 pg/mL), the levels of serum GRP78 were significantly (*p* < 0.005) higher in hens with early-stage OVCA (590.19 ± 198.18 pg/mL), and increased further in hens with late-stage OVCA (1261.38 ± 372.85) (*p* < 0.003) (Figure 7B). Thus, these results suggest that serum GRP78 levels increase in association with OVCA development and progression in patients and hens.

**Figure 8:**
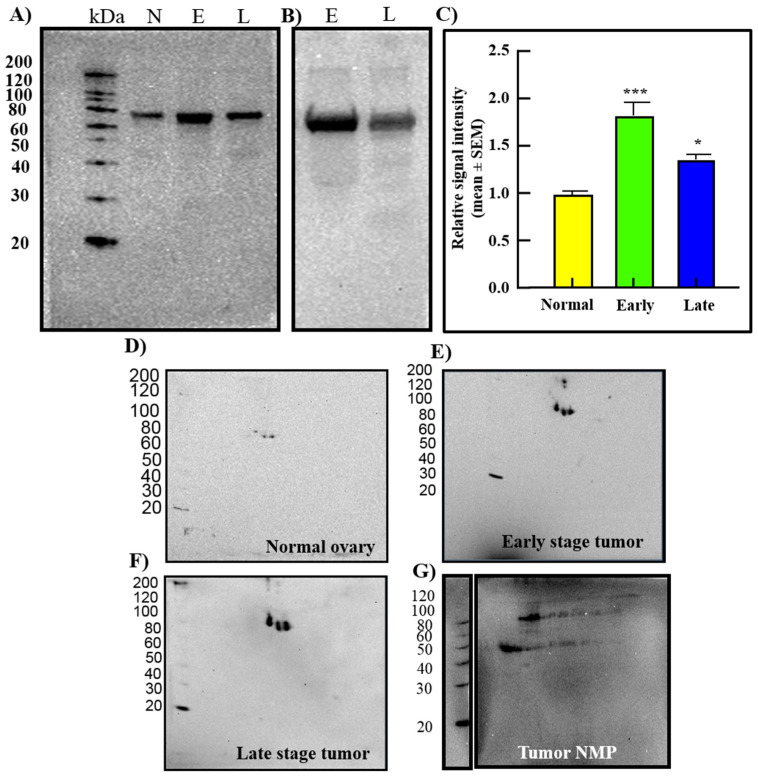
Detection of GRP78 protein by 1-dimensional and 2-dimensional Western blotting in normal ovary or ovaries with tumor in hens. (**A**) 1-D-WB detected GRP78 in normal ovary (N) and ovaries with early- (E) and late-stage (L) serous OVCA (*n* = 5 each). Compared with normal, strong expression of GRP78 is seen in early-stage and late-stage OVCA. (**B**) Homogenates of malignant cells from early (E) and late (L) stage HGSC showed strong immunoreactivities for GRP78. (**C**) Relative signal intensity of 1-D-WB bands shown in panels A and B, presented as a detected intensity ratio (mean ± SEM). (**D**–**F**) 2-D-WB using normal ovarian or tumor ovarian extracts showed specific staining for GRP78 (approximately 78 kDa) for normal (**D**), early-stage endometrioid (**E**) and late-stage serous (**F**) OVCA. (**G**) Nuclear matrix protein (NMP) from a late-stage HGSC also showed strong GRP78 staining in 2-D-WB. * *p* < 0.05, *** *p* < 0.001.

Compared with normal hens, 1-D-WB and 2-D-WB showed strong expression of GRP78 in tumor homogenates (total protein) from hens with OVCA at early and late stages (Figure 8A–F), and by tumor NMP (Figure 8G). A similar pattern of staining was shown by the cancer cells from early and late-stage OVCA in hens (Figure 8B,C) as was observed in patients (Figure 3). Thus, protein and gene expression data for GRP78 support immunohistochemical observations. Further, 1-D-WB of tumor NMP supports the nuclear staining of GRP78 observed in immunohistochemical studies.

### 3.4. Enhancement in the Signal Intensity of TVUS Scanning by GRP78-Targeted Imaging Agents for the Detection of Ovarian Tumors

**Figure 9:**
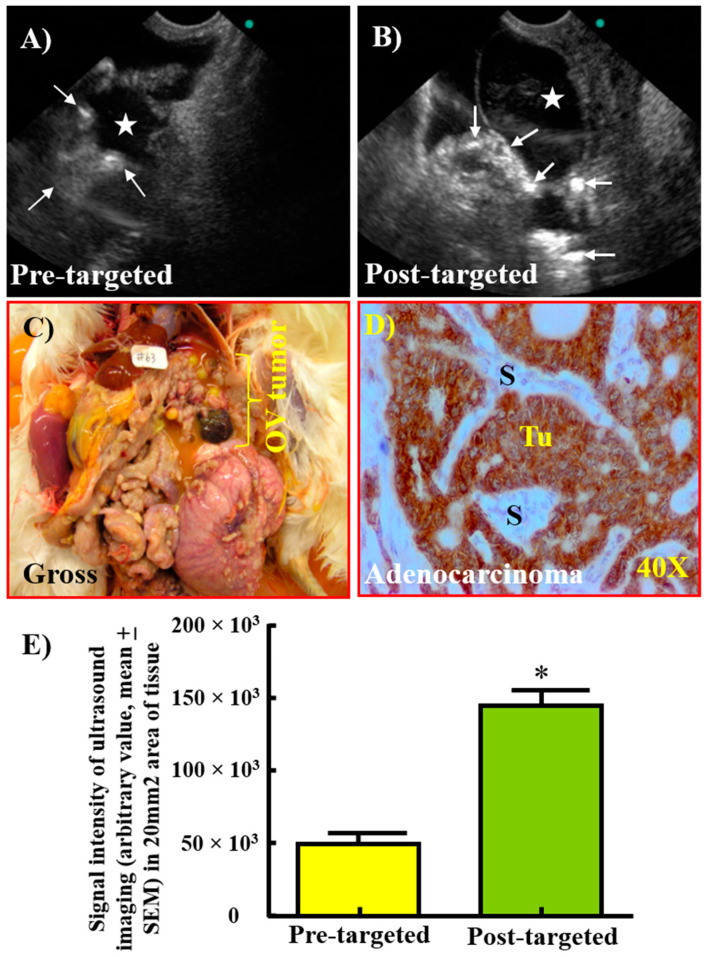
Enhancement in signal intensity of TVUS scanning by GRP78-targeted imaging of ovarian tumors in hens. (**A**) Pre-targeted scan of an ovary suspected to have solid mass (arrows) and ascites-like fluid (star). (**B**) Sonogram of the same ovary presented in (**A**) imaged after injection with GRP78-targeted imaging agents. Compared with pre-targeted imaging, post-targeted scan showed remarkable increase in imaging signals confirming the binding of imaging agents with the tumor (arrows). (**C**) Gross examination confirmed the presence of solid mass in the ovary accompanied with ascites. (**D**) Immunohistochemical examination showed intense expression of GRP78 by the serous tumor. (**E**) The intensity of ultrasound signals was significantly (*p* < 0.0001) higher in post-targeted imaging than pre-targeted imaging. OV = ovarian; S = stroma; Tu = tumor; 40× = Magnification. * *p* < 0.0001.

Compared with pre-targeted (43.31 × 10^3^ ± 10.7 × 10^3^), the signal intensities of GRP78-targeted TVUS imaging from ovarian tumors (145.00 × 10^3^ ± 15.4 × 10^3^) were significantly higher (*p* < 0.0001) (Figure 9). Gross examination of hens after targeted imaging confirmed the presence of tumor-associated mass in the ovary. Similar patterns of increase in signal intensities due to GRP78-targeted imaging were observed among different histological types of ovarian tumors. Immunohistochemical, Western blotting and gene expression studies for GRP78 expression supported the observations of GRP78-targeted-TVUS imaging. Therefore, targeted imaging agents bonded with their targets (GRP78) expressed on the surface of the malignant cells and enhanced the signal intensities of TVUS imaging for ovarian tumors.

## 4. Discussion

This is the first report describing the changes at the cellular and molecular levels of GRP78 (an endoplasmic reticulum resident protein and a marker of cellular stress) during OVCA development in patients and hens, as a preclinical model of spontaneous OVCAS. This is also the first report to show that GRP78-targeted imaging agents enhanced the resolution of traditional TVUS imaging. This study also showed increase in GRP78 levels during OVCA development. Similarities in expression of GRP78 during OVCA development in humans and hens suggest the suitability of laying hens for translational studies to develop an early detection test for OVCA based on GRP78-targeted imaging and its serum levels.

Information on the early molecular and cellular changes associated with OVCA development are limited, and understanding such factors involved in malignant changes are critical to establishing an early detection test and/or treatment of OVCA. This study showed that, compared with normal and benign ovarian tumors, the intensity of GRP78 expression was higher in ovarian malignant tumors at early and late stages. Similar findings were also observed in an immunohistochemical study on the expression of GRP78 in OVCA [29] as well as malignancies of several other organs [30,31,32,33]. Thus, these results suggest that increase in GRP78 expression may be an indicator of early changes associated with OVCA development. However, the reason(s) for increase in GRP78 expression and its association with OVCA development is (are) unknown. The Cancer Genome Atlas (TCGA) reports expression of GRP78 in several cancers, including ovarian, lung, breast, and skin cancers [34,35]. Further, increase in expression of GRP78 is associated with poor survival in renal carcinoma, as reported by the Human Protein Atlas [36]. Information on changes in GRP78 expression is critical for designing treatment intervention as well as early detection of HGSC, as observed in this study.

Longstanding chronic inflammation and cellular stress are hallmarks of malignant transformation [6,37,38]. The ovarian surface and the fimbria of the oviduct are constantly exposed to various bio-molecules associated with ovulation, and frequent ovulation has been suggested as a risk factor for OVCA [7]. Ovulation is an inflammatory event, and during ovulation the ovarian surface epithelium (site of ovulation) and the fimbrial surface epithelium (site of receiving of ovulated egg) are exposed to various factors including cytokines [39]. Furthermore, ovulation is followed by the influx of immune cells to these sites, leading to a chronic inflammatory state in the ovary. When the inflammatory condition continues and remains unresolved, further influx of immune cells results in an oxidative burst, leading to a hypoxic state and production of reactive oxygen species [7,12]. Under stressful environments, including hypoxic condition, cells enhance the secretion of GRP78 as a mechanism to withstand stress and survive [12]. Laying hens are frequent ovulators, and this study showed increased expression of GRP78 in hens with OVCA. Thus, it is possible that stressful conditions, including frequent ovulation, to which OSE cells and the fimbria of the fallopian tube are exposed, may lead to the increased expression of GRP78, which may be involved in malignant transformation in these tissues. However, the mechanism of GRP78-induced malignant transformation of the OSE or fimbria is unknown.

Following enhancement in expression under a stressful condition, GRP78 has been reported to have three main fates: one portion moves to the cell surface from the endoplasmic reticulum, another portion is secreted into the circulation, and the third portion translocates to the nucleus [40]. After translocating to the nucleus, GRP78 has been suggested to inhibit DNA-damage repair mechanisms [41], facilitating the growth of abnormal cells with altered or mutated DNA sequences. Uncontrolled growth of abnormal cells leads to the development of a malignant condition. In this study, both the cytoplasmic and nuclear staining of GRP78 were observed in ovarian malignant cells in women and hens. Moreover, in addition to the enhanced expression of GRP78 by malignant cells, serum levels of GRP78 also increased in association with OVCA development and progression in both patients and hens. These results suggest that GRP78 may be a potential serum marker for early OVCA detection. Furthermore, the portion of GRP78 expressed by the cell surface offers a potential target for imaging to detect OVCA at early stage by improving or enhancing the resolution of TVUS imaging. In this study, we developed, for the first time, a GRP78-targeted imaging agent, and this agent showed binding with its target and enhanced the signal intensity of traditional TVUS imaging from ovarian tumors in hens.

## 5. Conclusions

This study has several translational strengths. Enhanced expression of GRP78 by the cell surface during OVCA development can be detected by GRP78-targeted TVUS imaging. In addition, GRP78 becomes available in serum and is detectable by immunoassay. Thus, GRP78 has the potential to be a serum marker as well as an imaging target for the detection of OVCA at early stage. Moreover, OVCA in hens can be used to study and develop GRP78-targeted therapeutics, and to examine their effectiveness. As GRP78 has more than 90% homology between human and hens [42], information on GRP78 obtained from hen OVCA can be easily translated to humans. Thus, the laying hen represents a feasible model to study and establish an early detection test for OVCA using serum levels of GRP78 and GRP78-targeted TVUS imaging.

Smaller sample size is a limitation of this study. However, taken together, the results of this study showed that enhancement in GRP78 expression at molecular and cellular levels is associated with OVCA development and progression. GRP78 represents a potential target to be detected by targeted-TVUS imaging and a serum marker for early detection of OVCA. This study will serve as the foundation for a larger clinical study to establish the feasibility of GRP78 as a serum marker and imaging target for early detection of OVCA.

## Data Availability

All data for this study is contained within the article and the Supplementary Materials.

## Supplementary Materials

The following supporting information can be downloaded at https://www.mdpi.com/article/10.3390/cancers15041140/s1. Supplementary Figure S1. Immunohistochemical detection of GRP78 expression in normal (A) and BRCA1+ fimbria (B) of the fallopian tube. FSE = Fimbrial surface epithelial cell. S = Stroma, Arrows are examples of GRP78-expressing cells. Supplementary Figure S2. Immunohistochemical staining for GRP78 in ovarian endometriotic lesion (A) and late-stage high-grade serous carcinoma (HGSC) (B). Few cells in the epithelial layer of endometriotic lesion showed immunoreactivity for GRP78 expression. Sparse immunopositive staining is observed in endometriotic lesions while intense stain is observed in ovarian. S = Stroma, Tu = Tumor. Arrows are examples of GRP78-expressing cells. Supplementary Figure S3. Uncropped and unedited 1- and 2-dimensional Western blots for GRP78. For blots with samples not pertinent to this study, bands of interest are outlined in red.

## References

[1] Surveillance E., End Results (SEER) Program (2016). SEER*Stat Database: North American Association of Central Cancer Registries (NAACCR) Incidence Data-Cancer in North America (CiNA) Analytic File, 1995-2014.

[2] Howlader N., Krapcho M., Noone A.M., Miller D., Bishop K., Kosary C.L., Yu M., Ruhl J., Tatalovich Z., Mariotto A. SEER Cancer Statistics Review, 1975–2014. National Cancer Institute. Bethesda, MD. https://seer.cancer.gov/csr/1975_2014/.

[3] Torre L.A., Trabert B., DeSantis C.E., Miller K.D., Samimi G., Runowicz C.D., Gaudet M.M., Jemal A., Siegel R.L. (2018). Ovarian cancer statistics, 2018. CA Cancer J. Clin..

[4] Ries L.A. (1993). Ovarian cancer. Survival and treatment differences by age. Cancer.

[5] Pinsky P.F., Yu K., Kramer B.S., Black A., Buys S.S., Partridge E., Gohagan J., Berg C.D., Prorok P.C. (2016). Extended mortality results for ovarian cancer screening in the PLCO trial with median 15years follow-up. Gynecol. Oncol..

[6] Jaworek J., Leja-Szpak A., Nawrot-Porabka K., Szklarczyk J., Kot M., Pierzchalski P., Goralska M., Ceranowicz P., Warzecha Z., Dembinski A. (2017). Effects of melatonin and its analogues on pancreatic inflammation, enzyme secretion, and tumorigenesis. Int. J. Mol. Sci..

[7] Gonzalez F., Rote N.S., Minium J., Kirwan J.P. (2006). Reactive oxygen species-induced oxidative stress in the development of insulin resistance and hyperandrogenism in polycystic ovary syndrome. J. Clin. Endocrinol. Metab..

[8] Elssner A., Doseff A.I., Duncan M., Kotur M., Wewers M.D. (2004). IL-16 is constitutively present in peripheral blood monocytes and spontaneously released during apoptosis. J. Immunol..

[9] Chance B., Sies H., Boveris A. (1979). Hydroperoxide metabolism in mammalian organs. Physiol. Rev..

[10] Hendershot L.M., Valentine V.A., Lee A.S., Morris S.W., Shapiro D.N. (1994). Localization of the gene encoding human BiP/GRP78, the endoplasmic reticulum cognate of the HSP70 family, to chromosome 9q34. Genomics.

[11] Ames B.N., Gold L.S., Willett W.C. (1995). The causes and prevention of cancer. Proc. Natl. Acad. Sci. USA.

[12] Li Z., Li Z. (2012). Glucose regulated protein 78: A critical link between tumor microenvironment and cancer hallmarks. Biochim. Biophys Acta.

[13] Barua A., Bitterman P., Abramowicz J.S., Dirks A.L., Bahr J.M., Hales D.B., Bradaric M.J., Edassery S.L., Rotmensch J., Luborsky J.L. (2009). Histopathology of ovarian tumors in laying hens: A preclinical model of human ovarian cancer. Int. J. Gynecol. Cancer.

[14] Rodriguez-Burford C., Barnes M.N., Berry W., Partridge E.E., Grizzle W.E. (2001). Immunohistochemical expression of molecular markers in an avian model: A potential model for preclinical evaluation of agents for ovarian cancer chemoprevention. Gynecol. Oncol..

[15] Stammer K., Edassery S.L., Barua A., Bitterman P., Bahr J.M., Hales D.B., Luborsky J.L. (2008). Selenium-Binding Protein 1 expression in ovaries and ovarian tumors in the laying hen, a spontaneous model of human ovarian cancer. Gynecol. Oncol..

[16] Paris E.A., Bahr J.M., Bitterman P., Basu S., Abramowicz J.S., Barua A. (2021). Incidence of malignant transformation in the oviductal fimbria in laying hens, a preclinical model of spontaneous ovarian cancer. PLoS ONE.

[17] Barua A., Abramowicz J.S., Bahr J.M., Bitterman P., Dirks A., Holub K.A., Sheiner E., Bradaric M.J., Edassery S.L., Luborsky J.L. (2007). Detection of ovarian tumors in chicken by sonography: A step toward early diagnosis in humans?. J. Ultrasound Med..

[18] Barua A., Yellapa A., Bahr J.M., Adur M.K., Utterback C.W., Bitterman P., Basu S., Sharma S., Abramowicz J.S. (2015). Interleukin 16- (IL-16-) targeted ultrasound imaging agent improves detection of ovarian tumors in laying hens, a preclinical model of spontaneous ovarian cancer. Biomed. Res. Int..

[19] Barua A., Bradaric M.J., Kebede T., Espionosa S., Edassery S.L., Bitterman P., Rotmensch J., Luborsky J.L. (2007). Anti-tumor and anti-ovarian autoantibodies in women with ovarian cancer. Am. J. Reprod. Immunol..

[20] Barua A., Qureshi T., Bitterman P., Bahr J.M., Basu S., Abramowicz J.S. (2007). Molecular targeted imaging of vascular endothelial growth factor receptor (VEGFR)-2 and anti-NMP autoantibodies detect ovarian tumor at early stage. Cancer Res..

[21] Yu E., Lee H., Oh W., Yu B., Moon H., Lee I. (1999). Morphological and biochemical analysis of anti-nuclear matrix protein antibodies in human sera. J. Korean Med. Sci..

[22] Penumatsa K., Edassery S.L., Barua A., Bradaric M.J., Luborsky J.L. (2010). Differential expression of aldehyde dehydrogenase 1a1 (ALDH1) in normal ovary and serous ovarian tumors. J. Ovarian Res..

[23] Yellapa A., Bahr J.M., Bitterman P., Abramowicz J.S., Edassery S.L., Penumatsa K., Basu S., Rotmensch J., Barua A. (2012). Association of interleukin 16 with the development of ovarian tumor and tumor-associated neoangiogenesis in laying hen model of spontaneous ovarian cancer. Int. J. Gynecol. Cancer.

[24] Khan M.F., Bahr J.M., Yellapa A., Bitterman P., Abramowicz J.S., Edassery S.L., Basu S., Rotmensch J., Barua A. (2012). Expression of leukocyte inhibitory immunoglobulin-like transcript 3 receptors by ovarian tumors in laying hen model of spontaneous ovarian cancer. Transl. Oncol..

[25] Le Naour F., Brichory F., Misek D.E., Brechot C., Hanash S.M., Beretta L. (2002). A distinct repertoire of autoantibodies in hepatocellular carcinoma identified by proteomic analysis. Mol. Cell Proteom..

[26] Towbin H., Staehelin T., Gordon J. (1979). Electrophoretic transfer of proteins from polyacrylamide gels to nitrocellulose sheets: Procedure and some applications. Proc. Natl. Acad. Sci. USA.

[27] Yellapa A., Bitterman P., Sharma S., Guirguis A.S., Bahr J.M., Basu S., Abramowicz J.S., Barua A. (2014). Interleukin 16 expression changes in association with ovarian malignant transformation. Am. J. Obstet. Gynecol..

[28] Barua A., Yellapa A., Bahr J.M., Abramowicz J.S., Edassery S.L., Basu S., Rotmensch J., Bitterman P. (2012). Expression of death receptor 6 by ovarian tumors in laying hens, a preclinical model of spontaneous ovarian cancer. Transl. Oncol..

[29] Fan L.M., Su J., Dong H., Wei M., Cui M.H. (2013). Expression and role of glucose-regulated protein 78 in ovarian serous adenocarcinoma. Zhonghua Yi Xue Za Zhi.

[30] Shin B.K., Wang H., Yim A.M., le Naour F., Brichory F., Jang J.H., Zhao R., Puravs E., Tra J., Michael C.W. (2003). Global profiling of the cell surface proteome of cancer cells uncovers an abundance of proteins with chaperone function. J. Biol. Chem..

[31] Mintz P.J., Kim J., Do K.A., Wang X., Zinner R.G., Cristofanilli M., Arap M.A., Hong W.K., Troncoso P., Logothetis C.J. (2003). Fingerprinting the circulating repertoire of antibodies from cancer patients. Nat. Biotechnol..

[32] Davidson D.J., Haskell C., Majest S., Kherzai A., Egan D.A., Walter K.A., Schneider A., Gubbins E.F., Solomon L., Chen Z. (2005). Kringle 5 of human plasminogen induces apoptosis of endothelial and tumor cells through surface-expressed glucose-regulated protein 78. Cancer Res..

[33] Cali G., Insabato L., Conza D., Bifulco G., Parrillo L., Mirra P., Fiory F., Miele C., Raciti G.A., di Jeso B. (2014). GRP78 mediates cell growth and invasiveness in endometrial cancer. J. Cell Physiol..

[34] The Cancer Genome Atlas. https://gdc.cancer.gov/.

[35] Grossman R.L., Heath A.P., Ferretti V., Varmus H.E., Lowy D.R., Kibbe W.A., Staudt L.M. (2016). Toward a Shared Vision for Cancer Genomic Data. N. Engl. J. Med..

[36] The Human Protein Atlas. https://www.proteinatlas.org/.

[37] Coussens L.M., Werb Z. (2002). Inflammation and cancer. Nature.

[38] Calaf G.M., Urzua U., Termini L., Aguayo F. (2018). Oxidative stress in female cancers. Oncotarget.

[39] Machelon V., Emilie D. (1997). Production of ovarian cytokines and their role in ovulation in the mammalian ovary. Eur. Cytokine Netw..

[40] Ni M., Zhang Y., Lee A.S. (2011). Beyond the endoplasmic reticulum: Atypical GRP78 in cell viability, signalling and therapeutic targeting. Biochem. J..

[41] Barker S., Weinfeld M., Zheng J., Li L., Murray D. (2005). Identification of mammalian proteins cross-linked to DNA by ionizing radiation. J. Biol. Chem..

[42] National Center for Biotechnology Information, U.S. National Library of Medicine Endoplasmic Reticulum Chaperone BiP Precursor [Gallus Gallus]. https://blast.ncbi.nlm.nih.gov/Blast.cgi.

