# Supplementary material for: Glucose-Regulated Protein 78 Is a Potential Serum and Imaging Marker for Early Detection of Ovarian Cancer
Source: Cancers (Basel). 2023 Feb 10;15(4):1140. doi: 10.3390/cancers15041140 (PMC9954741; doi:10.3390/cancers15041140)
Supplement: Supplementary file 1 [file cancers-15-01140-s001.zip › cancers-2140570-supplementary.pdf]

cancers
MDPI

Supplementary Materials: Glucose-Regulated Protein 78 Is a Potential Serum and Imaging Marker for Early Detection of Ovarian Cancer

Elizabeth A. Paris, Janice M. Bahr, Jacques S. Abramowicz, Sanjib Basu and Animesh Barua

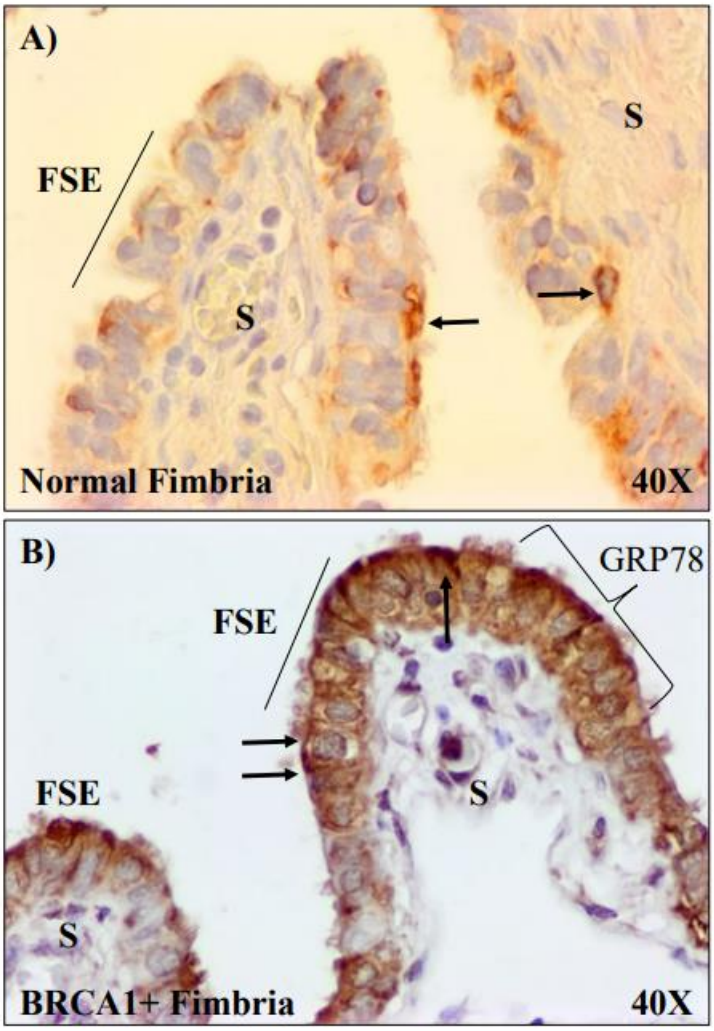

Figure S1. Immunohistochemical detection of GRP78 expression in normal (**A**) and BRCA1+ fimbria (**B**) of the fallopian tube. FSE = Fimbrial surface epithelial cell. S = Stroma, Arrows are examples of GRP78-expressing cells.

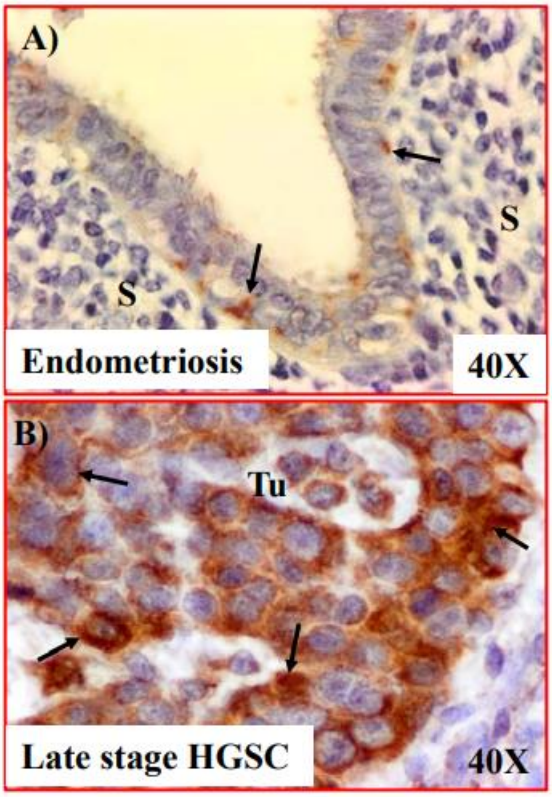

Figure S2. Immunohistochemical staining for GRP78 in ovarian endometriotic lesion (**A**) and late-stage high-grade serous carcinoma (HGSC) (**B**). Few cells in the epithelial layer of endometriotic lesion showed immunoreactivity for GRP78 expression. Sparse immunopositive staining is observed in endometriotic lesions while intense stain is observed in ovarian. S = Stroma, Tu = Tumor. Arrows are examples of GRP78-expressing cells.

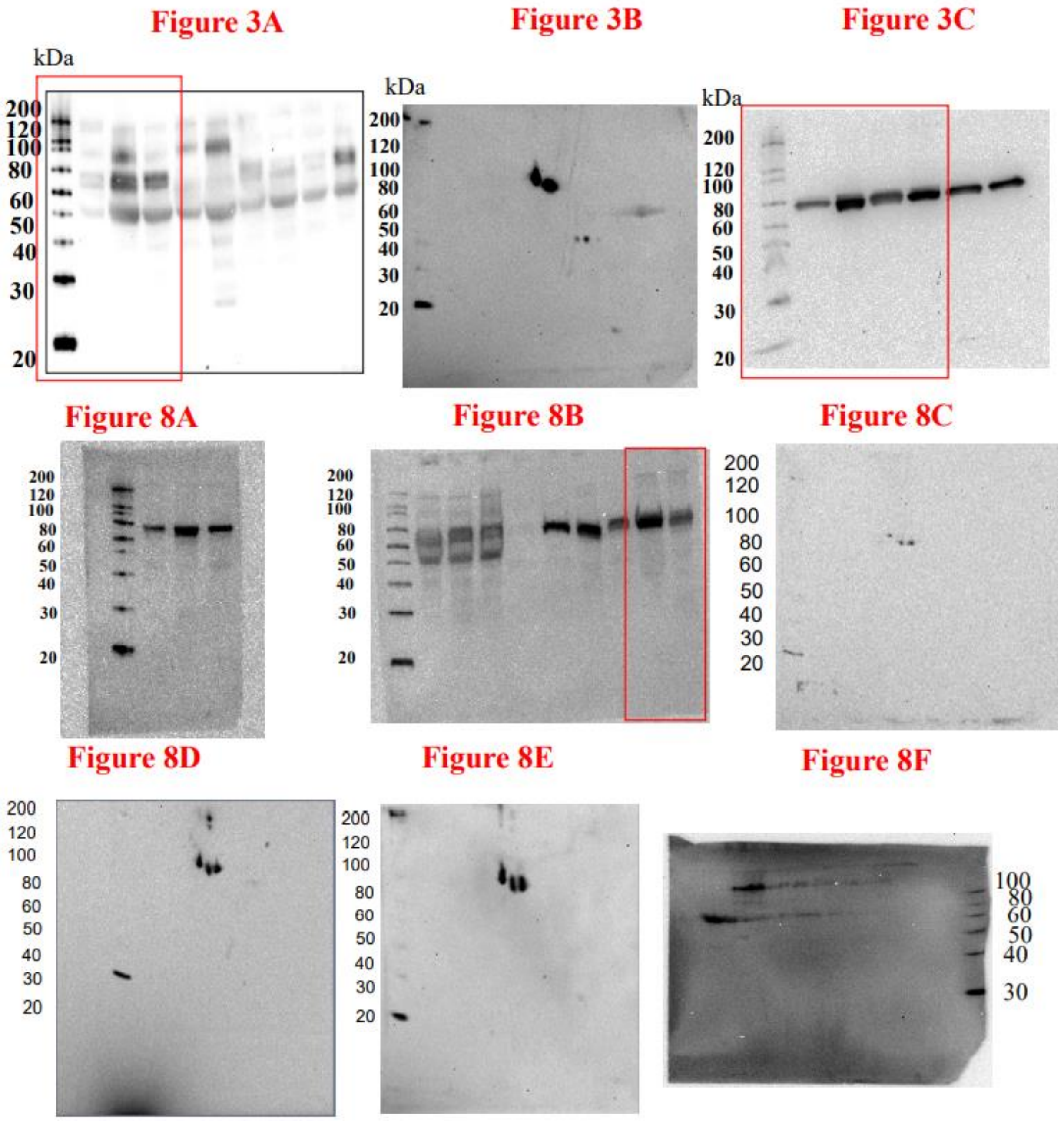

Figure S3. Uncropped and unedited 1- and 2-dimensional Western blots for GRP78. For blots with samples not pertinent to this study, bands of interest are outlined in red.
